# *In Vivo* Pharmacokinetics/Pharmacodynamics of Cefquinome in an Experimental Mouse Model of *Staphylococcus Aureus* Mastitis following Intramammary Infusion

**DOI:** 10.1371/journal.pone.0156273

**Published:** 2016-05-24

**Authors:** Yang Yu, Yu-Feng Zhou, Mei-Ren Chen, Xiao Li, Gui-Lin Qiao, Jian Sun, Xiao-Ping Liao, Ya-Hong Liu

**Affiliations:** 1 National Risk Assessment Laboratory for Antimicrobial Resistance of Animal Original Bacteria, South China Agricultural University, Guangzhou, Guangdong, China; 2 Guangdong Provincial Key Laboratory of Veterinary Pharmaceutics, Development and Safety Evaluation, South China Agricultural University, Guangzhou, Guangdong, China; 3 Defense Threat Reduction Agency, 8725, John J Kingman Rd, MS 6201, Ft Belvoir, Virginia, 22060–6201, United States of America; University of South Dakota, UNITED STATES

## Abstract

*Staphylococcus aureus* remains the major cause of morbidity of bovine mastitis worldwide leading to massive economic losses. Cefquinome is a fourth generation cephalosporin, which preserves susceptibility and antibacterial activity against *S*. *aureus*. This work aims to study the pharmacokinetic (PK) and pharmacodynamic (PD) modeling following intramammary administration of cefquinome against *S*. *aureus* mastitis. The mouse model of *S*. *aureus* mastitis was developed for the PK/PD experiments. The plasma PK characteristics after intramammary injection of cefquinome at various single doses of 25, 50, 100, 200, 400 μg per gland (both fourth pairs of glands: L4 and R4) were calculated using one-compartment and first-order absorption model. PD study was investigated based on twenty-one intermittent dosing regimens, of which total daily dose ranged from 25 to 4800 μg per mouse and dosage intervals included 8, 12 or 24 h. The sigmoid *E*_*max*_ model of inhibitory effect was employed for PK/PD modeling. The results of PK/PD integration of cefquinome against *S*. *aureus* suggested that the percentage of duration that drug concentration exceeded the minimal inhibitory concentration (%T>MIC) and the ratio of area under time-concentration curve over MIC (AUC/MIC) are important indexes to evaluate the antibacterial activity. The PK/PD parameters of %T>MIC and AUC_0-24_/MIC were 35.98% and 137.43 h to obtain a 1.8 logCFU/gland reduction of bacterial colony counts *in vivo*, against *S*. *aureus* strains with cefquinome MIC of 0.5μg/ml.

## Introduction

Bovine mastitis is an inflammation of the mammary glands usually resulted from bacterial colonization, consequence of yeast and even fungal or algae infection [1]. It can produce significant economic losses to the dairy industry due to quality deterioration of milk, dedication and veterinary care expenses and prohibitive labor costs for producers [1]. According to the clinical features, intramammary infection (IMI) is classified into two types: clinical mastitis and subclinical mastitis. Clinical mastitis is acute and severe, and may end up with death of patients. Subclinical mastitis (also called chronic mastitis) is rarely lethal but capable of resulting in a vast amount of financial losses. *Staphylococcus aureus* is the primary pathogen responsible for both forms of mastitis. Treatment of *S*. *aureus* mastitis is greatly difficult because the pathogen can release exotoxin, be resistant to many antimicrobials frequently, and survive in the intracellular space where the drug concentration is often low [1].

Cefquinome is a fourth generation cephalosporin applied as veterinary medicine solely. Cefquinome is stable to common plasmid- and chromosomally mediated beta-lactamases exhibiting antibacterial activity against a broad spectrum of Gram-positive and Gram-negative bacterial species. Most *S*. *aureus* isolates from bovine mastitis are susceptible to cefquinome [2–4]. The pharmacokinetic (PK) characteristics of cefquinome have been studied in various animals, such as, sheep, goats, cattle, buffalo calves, and camels via intravenous (i.v.) or intramuscular (i.m.) administration [5–9]. The PK profiles of cefquinome after local intramammary administration have also been performed in lactating cows and buffalo [10–12]. Previous report suggests that intramammary recipe is more successful than the systemic therapy, especially for *Staphylococcal* mastitis with a considerable microbiological cure rate [13,14]. The integration of PK/PD model has been widely applied in evaluation of antibacterial activity and optimization of dosing regimens. Although the PK fate of cefquinome and its efficacy of clinical treatment have been widely studied, there is no complete research linking the PK parameters to the PD effectiveness on mastitis therapy following various intramammary administration dosing regimens.

In the present study, we adopted the mouse model of *S*. *aureus* mastitis (MMSAM), which is popular in investigating the IMI and the treatment as an alternative model rather than practicing in target animal of cows [15]. This is because the costs related to the experimental IMI in cows are prohibitively high even to achieve the minimal power of statistical analysis [16]. Besides, the team has well studied this model through different aspects like pathology and application [16]. It is undeniable that possible differences between IMIs in two species, however results from this model may bring to light important mechanisms that could also take place during bovine mastitis caused by *S*. *aureus*. The objective of our experiment was to integrate PK/PD features and estimate the prime values of PK/PD parameters required for different levels of antibacterial efficacy.

## Materials and Methods

### Antimicrobial agents

Sterile powder of cefquinome for injection was purchased from Qilu Animal Health Products CO., LTD, Shandong, China. Stock solution of cefquinome was prepared in sterile water at 40,000 μg/ml and stored at -20°C till use. Working solutions were prepared daily by appropriate dilution of the stock solution with stroke-physiological saline solution (SPSS) and ultrapure water, respectively.

### Bacterial strains and animals

*S*. *aureus* Newbould 305 (ATCC 29740), a mastitis isolates, was employed as the standard strain for experimental IMI of cows [17]. Thirty-eight *S*. *aureus* isolated from clinical bovine mastitis individuals in Inner Mongolia, China were also evaluated in this study. Broth and agar of Brain-Heart-Infusion (BHI), Mueller-Hinton (MH) and Mannitol Salt (MS) were purchased from Guangdong Huankai Microbial Sci. & Tech. CO., Ltd, Guangzhou, China.

All the lactating mice purchased from Vital River Laboratories, Beijing, China, with body weight ranged from 35 to 45 g, were bred in special-pathogen-free (SPF) environment, each two in one cage, with a 12: 12 light: dark circle and fed of SPF food (purchased from Southern Medical University, Guangzhou, China). All animal studies were approved by the Animal Use and Care Committee of South China Agricultural University and the guidelines of American Association for Accreditation of Laboratory Animal Care (AAALAC) were respected or followed during all the *in vivo* procedures [18].

### MIC test

Susceptibility tests were determined according to Clinical and Laboratory Standards Institute (CLSI) guideline [19]. The MIC_50_ and MIC_90_ values were calculated, which represented the MIC value inhibiting the growth of at least corresponding 50% and 90% of isolates in a test population [20]. Triplet MIC tests were performed for all the strains and mean value of MIC was used for data analysis.

### *In vitro* time-killing curves

*An overnight culture of S*. *aureus* Newbould 305 [17] was 10-fold diluted appropriately. Pathogens were exposed to five different cefquinome concentrations of 0.5×, 1×, 2×, 4× and 8× MIC and bred at 37°C with 200 rpm per minute shaking. Antibacterial activity against 2 initial inoculum of 10^6^ and 10^7^ CFU/mL were evaluated, respectively. Samples examined at 0, 3, 6, 9, and 24 h were subjected to 10-fold serial dilution and then plated onto MH agar for visible counts calculation. The detection limit was 100 CFU/ml. All the MH agar plates were cultured at 37°C for 22 to 24 h before colony counting.

### Pharmacokinetics

The PK trials were performed in healthy CD-1 mice lactating for 10–12 days (six mice for each group). As we know, mouse has five pairs of mammary glands, three pairs on the thorax and two on the abdomen, which are identified by a letter and a number indicating their relative anatomic location from head to tail. Mastitis model are usually performed on L4 (fourth on the left) and R4 (fourth on the right) abdominal glands, because of their biggest size and easily to be harvested. Intramammary administration of a single dose cefquinome of 25, 50, 100, 200 or 400 μg/gland was injected into the L4 and R4 glands’ canals of mouse through a tiny cut at the end of teat using a 32-gauge blunt needle. Blood samples (about 50 μl at each time) were harvested by retro-orbital puncture at the following time points: 5 min, 10 min, 15min, 0.5 h, 0.75 h, 1 h, 2 h, 3 h, 4 h, 6 h, 8 h, and 12 h after administration. Plasma samples were obtained after centrifuging at 4000 rpm for 10 min. The drug concentrations in plasma were determined using the high-performance liquid chromatography—Electro-Spray Ionization—Mass Spectrometry (HPLC-ESI-MS/MS) method reported previously [21]. The linearity of cefquinome quantitation was within a range of 0.01–2 μg/ml and correlation coefficients were above 0.9999. The extraction recoveries of cefquinome from plasma was >90%, and coefficients of variation were <10% for both withinruns and between runs. The limit of quantification (LOQ) and detection (LOD) were 0.01 and 0.005 μg/ml, respectively. The PK parameters were calculated by the Winnonlin software (version 5.2.1, Pharsight, St. Louis, MO, USA), including half-lives of first-order absorption (T_1/2ka_) and elimination (T_1/2Kel_), area under time-concentration curve (AUC), the peak plasma concentration (C_max_), and the time of maximum concentration (T_max_).

The gland tissue samples were collected and mammary gland concentrations were tested, which data will be published separately.

### The mouse model of *S*. *aureus* mastitis

The experimental conditions for mastitis model adopted here were similar to these as previously reported with minor modifications [16]. Briefly, 1 h following removal of 10–12 day-old offspring, lactating CD-1 mice (Vital River Laboratories, Beijing, China) were anaesthetized by intraperitoneal injection of 0.1 ml pentobarbital sodium (1.5%). A small cut at the end of teats was made to expose the mammary ducts and a 100 μl bacterial suspension containing about 4 × 10^3^ CFU of *S*. *aureus* Newbould 305 (it should be noticeable that this final selection of bacterial inoculation is tailored to our pathogen isolates and animals used according to bacteriological, clinical and pathological evaluations) was injected through the teat canal using a 32-gauge aseptic blunt needle. The bacterial suspension was prepared from a serial dilution of an overnight culture in BHI broth. Control animals were inoculated with SPSS at the same volume. After inoculating, a 9 h incubation is required to allow the growth of bacterium to log phase. Then the mice were sacrificed and the L4 and R4 glands were aseptically harvested and homogenized in 3 ml SPSS to calculate the bacterium counts. The tissue suspension were evaluated for bacterial counts on MSA plates after serial 10-fold dilutions. The detection limit was 300 CFU/gland. The criterion of full preparation of MMSAM was the amount of bacteria reaching 10^6−7^ CFU/gland, which is largely based on clinical and pathological evolutions. The number of animal ranged from 2 to 4 according to the different experiments.

### *In vivo* growth and time-killing curves

Totally 180 mice were divided into five groups, growth control and four treatment groups of various regimens. Following the preparation of MMSAM, a single dose of 50, 100, 200, 400 μg/gland (i.e. 100, 200, 400, 800 μg per mouse) was intramammary administrated, respectively (recording as 0 h). Control group was treated with sterile SPSS. For this module, four mice were sacrificed and tested for bacterial CFU counts (i.e. 8 mammary glands) at each time point of 0, 1, 3, 6, 9, 12, 24 h, 48 h and 72 h.

### PD experiments

*S*. *aureus* Newbould 305 was employed to determine the PD characteristics. After establishment of MMSAM, tested mice were divided into twenty-one groups treated with cefquinome under various dosing regimens. The dosage two-fold increased from 12.5 to 800 μg/gland and the dosing intervals were 8, 12 and 24 h (once, twice and thrice a day), respectively. Control group was treated with sterile SPSS. After the 24 h treatment, 4 mice a group (i.e. n = 8 for gland) of each dosing regimen were euthanized. The L4 and R4 glands were then harvested for bacterial CFU counts. The control group was sacrificed before the intramammary administration and at 24 h.

### PK/PD analysis

The PK/PD parameters of the 21 regimens were extrapolated from the corresponding single dosing PK data obtained above. The surrogate markers of antibacterial efficacy included the ratio of area under the concentration time curve to the MIC for 0 to 24 h (AUC_0-24_/MIC), the duration of drug concentration exceeding the MIC (%T > MIC) and the peak concentration divided by the MIC (C_max_/MIC). MIC_90_ value was employed for formulation of PK/PD indexes. The %T>MIC, AUC_0-24_, and C_max_ for multiple dosing regimens are calculated using following eq 1:

$$C_{n}=\frac{K_{a}FX_{0}}{\text{V}\left(K_{a}-K_{el}\right)}\left(\frac{1-e^{-nK_{el}\tau }}{1-e^{-K_{el}\tau }}\cdot e^{-kt}-\frac{1-e^{-nK_{a}\tau }}{1-e^{-K_{a}\tau }}\cdot e^{-K_{a}t}\right)$$ (1)

Where C is the concentration drug concentration, n is the dosing times, K_a_ is the absorption half-life, F is the bioavailability, V is the apparent volume of distribution, X_0_ is the dose of antibiotic, K_el_ is the elimination half-life and τ is the dosing interval. The PK parameter of K_a_, K_el_, and F/V are achieved in PK test mentioned above.

### PK/PD integration

The antimicrobial effect of cefquinome was analyzed by applying the sigmoid E_max_ model of inhibitory effect, as previously reported [22], which is defined as eq 2:

$$\text{E}=E_{max}-\frac{\left(E_{max}-E_{0}\right)\times C_{e}^{N}}{EC_{50}^{N}+C_{e}^{N}}$$ (2)

where *E* is the antibacterial effect, measured as the change in the bacterial counts (logCFU/gland) in the gland sample after 24 h of treatment compared to the initial colony counts; *E*_*max*_ is the ΔlogCFU_24h_ in the drug-free control sample; *E*_*0*_ is the ΔlogCFU_24h_ in the test sample containing cefquinome, when the maximum antibacterial effect was achieved; *C*_*e*_ is the PK/PD index (AUC_0-24_/MIC, C_max_/MIC or %T > MIC for serum drug concentration); *EC*_*50*_ is the value of PK/PD index of drug producing 50% of the maximum antibacterial effect; and N is the Hill coefficient, which describes the steepness of the effect curve resulting from each PK/PD indices.

## Results

### MICs of cefquinome against S. aureus Newbould 305 and mastitis isolates

Cefquinome MICs were 0.5 μg/ml against *S*. *aureus* Newbould 305 and 0.25 to 0.5 μg/ml against the thirty-eight isolates, with MIC_50_ and MIC_90_ both of 0.5 μg/ml (S1 Table).

### In vitro time killing curves of cefquinome against different inoculum load

**Fig 1:**
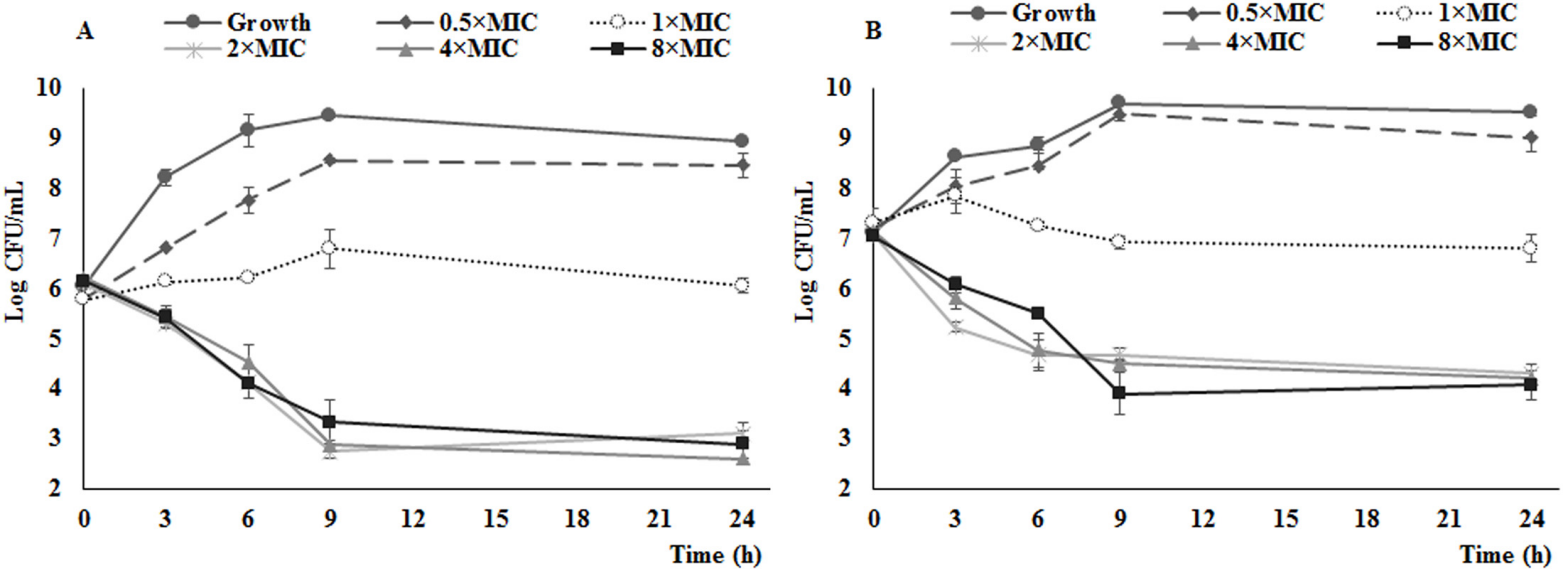
*In vitro* time-killing curves of cefquinome against *S*. *aureus* Newbould 305 with cefquinome MIC of 0.5 μg/ml. The cefquinome concentrations of 2×, 4×, and 8×MIC exhibited a same killing speed and actability, by reducing 2.5–3 logCFU/ml of bacterial colony during 9 h incubation. A and B shows different antibacterial activity against different initial bacterial load (10^6^ log_10_CFU/mL and 10^7^ log_10_CFU/mL). Each symbol represents mean value and bars represent standard deviations.

The in vitro time-killing curves against *S*. *aureus* Newbould 305 is presented in Fig 1. The killing profile of cefquinome showed low correlation with the exposed dose, as the killing speed did not change with the increasing drug concentration. At 2× MIC and all higher concentrations, bactericidal activity of 3.5 and a less than 3 logCFU/ml kill were observed against 10^6^ initial inoculum group and 10^7^ initial inoculum group, respectively. Cefquinome concentration less than MIC cannot inhibit the bacterial growth.

### Plasma PK of cefquinome

**Fig 2:**
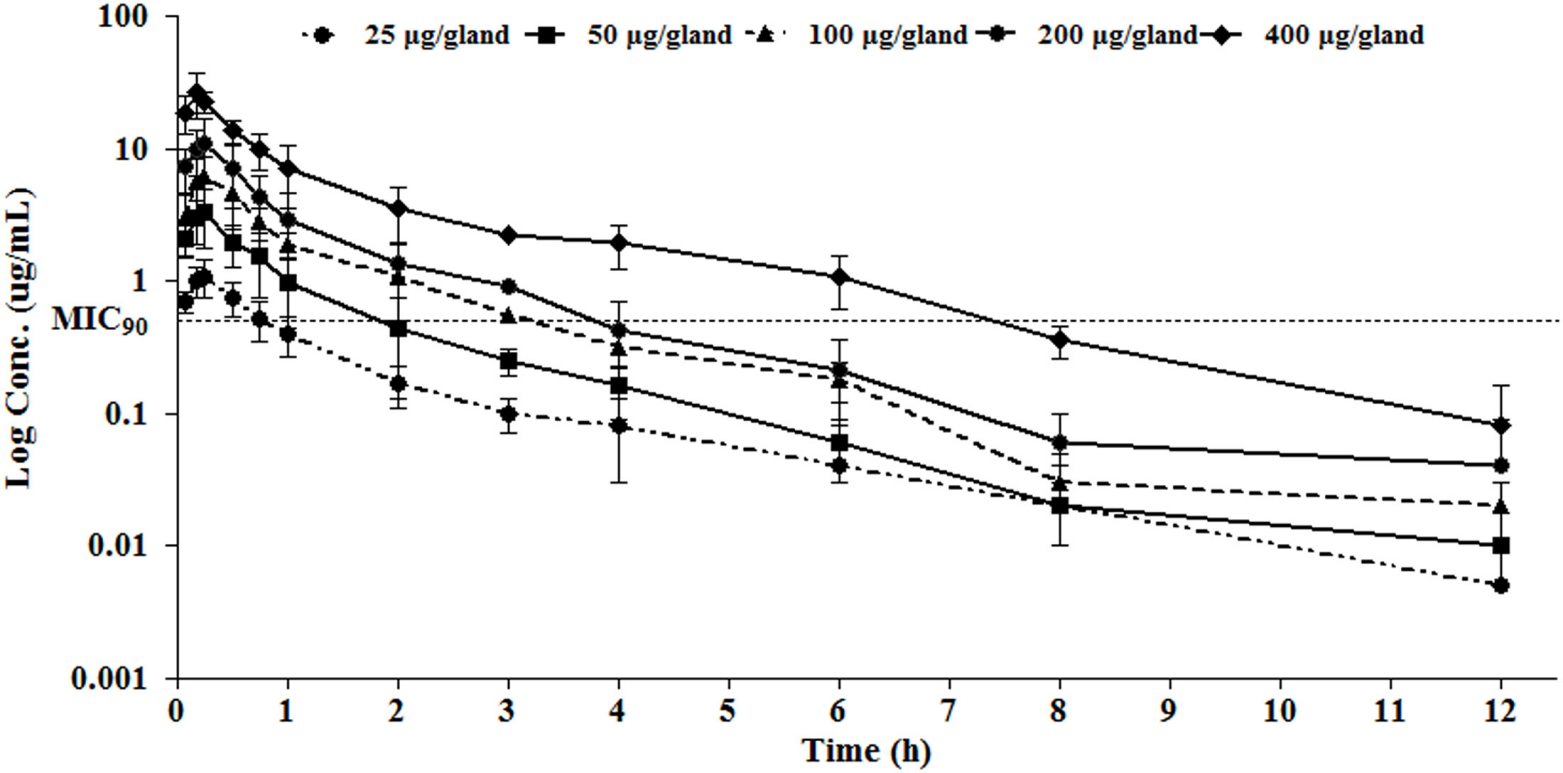
Semi-logarithmic plot of serum concentration versus time of cefquinome mouse model of *S*. *aureus* mastitis (n = 6), following a single intramammary administration dose of 25, 50, 100, 200, or 400 μg/gland. Bars represent standard deviations. Horizontal dotted line represents the MIC_90_ value of 38 isolates.

No adverse effects (including death of stress, acute death, depression, and abnormal behavior) were observed after intramammary administration. The plasma drug concentration data are considered largely for free drug since the low protein binding rate (17% in the mouse) [23] and the way we handle the sample preparation protein precipitation. The semi-logarithmic plots of plasma concentration-time curves for various dosages are shown in Fig 2. The one-compartment model with first-order absorption was the best-fit model to calculate the relevant PK parameters, as the Akaike Information Criterion (AIC) was the lowest. The T_max_ ranged from 0.17 to 0.27 h with a mean value of 0.22 h. T_1/2Kel_ of plasma varied from 0.34 to 0.49 h with an average of 0.4 h. C_max_ and AUC increased with dosage linearly (Table 1).

**Table 1:** PK parameters of cefquinome in plasma after intramammary administration analyzed by one-compartment model with first-order absorption (n = 6).

Variable(units)	Intramammary administration dose (μg/gland)					
	25	50	100	200	400	Mean ± SD
*T*_1/2Ka_ (h)	0.07±0.01	0.07±0.02	0.11±0.04	0.08±0.02	0.05±0.01	0.08 ± 0.02
*T*_1/2Kel_ (h)	0.49±0.08	0.41±0.1	0.34±0.12	0.35±0.06	0.39±0.07	0.4 ± 0.06
*T*_max_ (h)	0.22±0.02	0.21±0.03	0.27±0.03	0.22±0.02	0.17±0.02	0.22 ± 0.03
AUC (μg·h/ml)	0.99±0.08	2.55±0.31	4.93±0.55	8.03±0.52	18.57.28±1.9	NA
*C*_max_ (μg/ml)	1.03±0.04	3.02±0.17	5.83±0.32	10.29±0.33	24.33±1.02	NA

*T*_1/2Ka_, absorption half-life; *T*_1/2Kel_, elimination half-life; AUC, area under plasma concentration-time curve of 0 to 4 h; *T*_max_, time of maximum concentration; *C*_max_, maximum concentration; (n = 6).

### Establishment of MMSAM and in vivo time-killing curves

Acute clinical mastitis of bacterial colony counts reaching 10^7^ CFU was achieved by intramammary injection of 100 μl suspension containing about 4000 CFU and incubation for 9 h. The steady phase of colonization about 10^9^ CFU was found during 24 h and 48 h incubation (S1 Fig).

**Fig 3:**
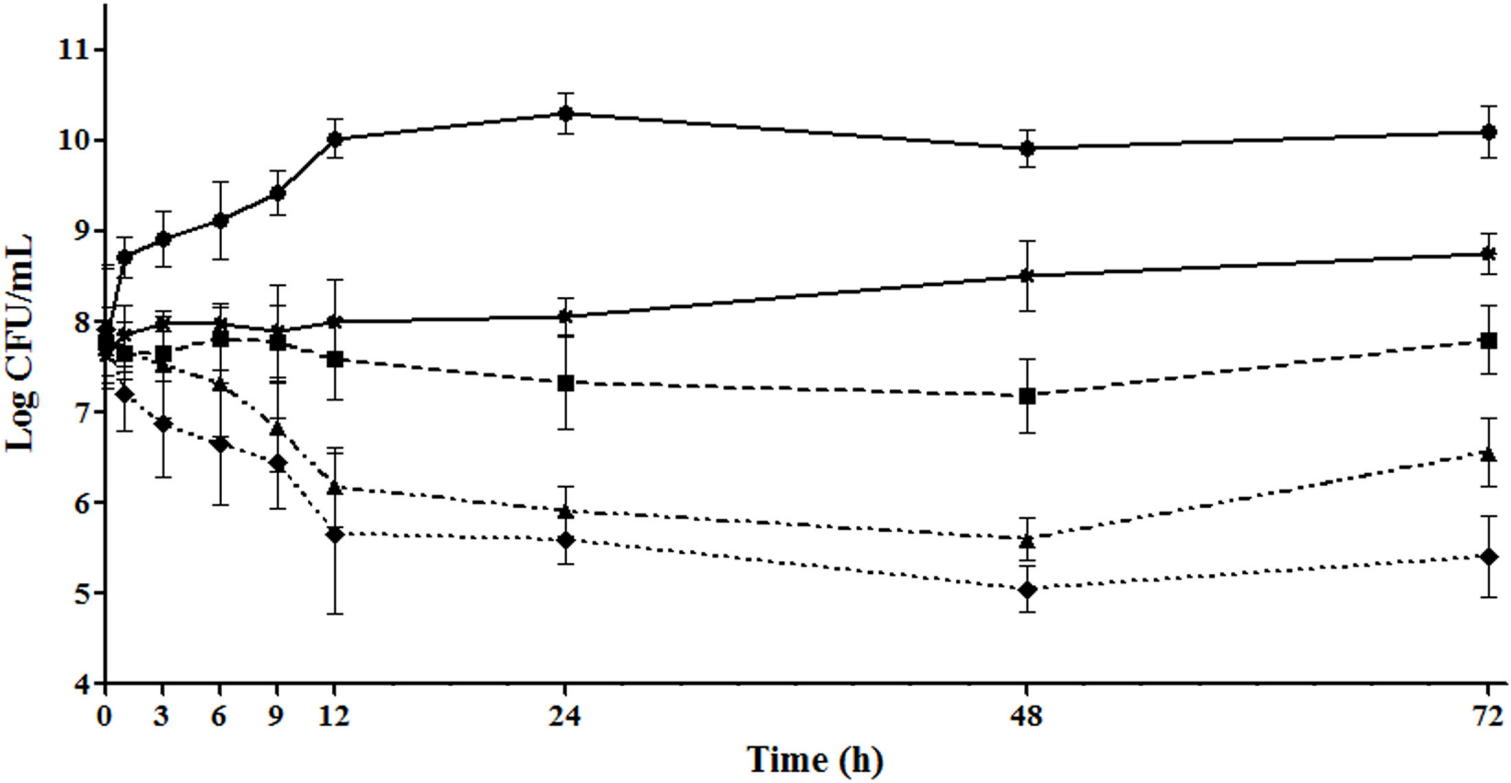
Changes in viable cell density (CFU/gland) of *S*. *aureus* and concentrations of antibiotics (×MIC) *in vivo* following a single treatment with cefquinome. Testing dosing regimens were single doses at 50, 100, 200 and 400 μg/gland by intramammary administration, (n = 4 for mice, i.e. for glands n = 8).

The *in vivo* time-killing course are shown in Fig 3. In the control group, initial colony counts was 7.76 logCFU/gland and increased to 10.29 logCFU/gland at 24 h. The killing speed of cefquinome in MMSAM was slower than that in broth medium *in vitro*. At dose of 200 and 400 μg/gland, an antibacterial activity, about 2.3 logCFU/gland reduction was observed after 24 h incubation, while no net change of bacteria load at dose of 50 and 100 μg/gland cefquinome was observed at 24 h. After a single dose, bacterial regrowth was observed in the four groups at 72 h.

### PD effectiveness of 21 dosing regimens

The treatment activity of cefquinome was evaluated by the net change of bacterial counts (logCFU/gland) during 24 h in MMSAM. Before administration, strains’ population reached 7.61 logCFU/gland (mean value) in the inoculated mammary. The bacterial load at 24 h after treatment were shown in S2 Fig. When given a dose above 400 μg/gland and with 8 or 12 h dosing intervals, a better antibacterial activity was observed with more than 2 logCFU/gland reduction versus 1 log for 200 μg/gland (P<0.05, two-tailed t-test). As the dose level and the dosing interval increased, the *in vivo* antibacterial activity of cefquinome was elevated, exhibiting a declining trend of survival strains’ population by the end of the experimental circle.

### PK/PD Integration

The PK/PD parameters of multiple dosing regimens are reported in S2 Table, regarding the regimens in PD experiments for which no kinetics were determined.

**Fig 4:**
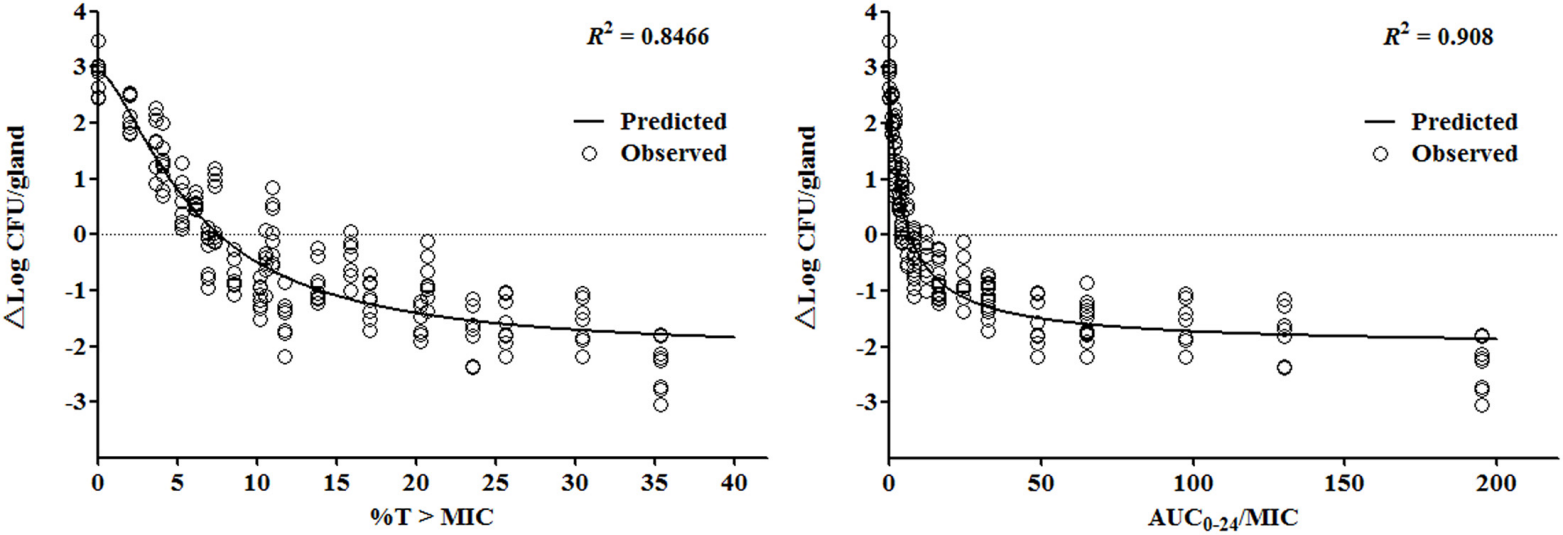
Sigmoid E_max_ relationships between *in vivo* antibacterial effect (△logCFU_24h_/gland) and PK/PD indexes of %T>MIC and AUC_0-24_/MIC against *S*. *aureus* Newbould 305. The lines represent the model fits of the data. *R*^*2*^ is the correlation coefficient.

The PK/PD profiles of plasma concentrations versus antibacterial effect were analyzed by the sigmoid *E*_*max*_ model of inhibitory effect (Fig 4). The correlation coefficient (*R*^*2*^) between antibacterial effects and %T>MIC and AUC_0-24_/MIC were 0.8466 and 0.908, accordingly. The PD parameters of *E*_*0*_, *E*_*max*_, PK/PD parameters required for various degrees of antibacterial activity and the Hill coefficient *N* are presented in Table 2.

**Table 2:** Integration of PK/PD after intramammary administration of cefquinome in mouse model of *S*. *aureus* mastitis.

Parameter	Value (Mean ± SD)	
	%T>MIC	AUC/MIC
Log *E*_max_ (logCFU/gland)	2.94±0.19	3.00±0.15
Log *E*_0_ (logCFU/gland)	-2.11±0.22	-2.04±0.13
Log *E*_max_—Log *E*_0_ (logCFU/gland)	5.05±0.33	5.03±0.22
*EC*_*50*_ (h)	6.12±0.49	4.44±0.45
For bacteriostatic action (h)	7.59±0.03	6.91±0.10
For 1.8 logCFU reduction (h)	35.98±0.03	137.43±0.10
Slope (*N*)	1.54±0.19	0.88±0.07

*E*_max_, the difference in the bacterial number in the control sample (drug-free) after 24 h incubation from initial inoculum (△logCFU_24h_/gland); *E*_0_, △logCFU_24h_/gland in the test sample containing cefquinome after 24 h incubation when the best antibacterial activity is reached; *EC*_*50*_, the value of PK/PD parameters when the half effect is achieved; *N*, the Hill coefficient.

### PK/PD Model Parameter Estimates for the Target Efficacy

The target values of cefquinome necessary to produce a bacteriostatic action and a 1.8-log_10_-CFU/gland reduction were 7.59% and 35.98% for %T>MIC and 6.91 and 137.43 h for AUC_0-24_/MIC, respectively.

## Discussion

In present study, the MICs of cefquinome against *S*. *aureus* Newbould 305 and clinical bovine mastitis isolates ranged from 0.25 to 0.5 μg/ml, which are in line with the values previously reported [23]. As a fourth-generation of cephalosporin, cefquinome maintain a remarkable antibacterial potential, since different species or strains causing mammary inflammation and gland tissue damages express the susceptibility to this drug in general [2–4]. Besides, we demonstrated that cefquinome was fairly effective, causing an over 3-log_10_-unit reduction of bacterial load in time-killing curves *in vitro*, suggesting a bactericidal activity of cefquinome.

In addition, several issues need to be illustrated. Firstly, according to PK study the absorption half-life (*t*_1/2ka_) of 0.09 ± 0.03 h was slightly shorter than 0.14 h for i.m. and 0.29 h for s.c. in beagle dogs [21]. After intramammary administration, a rapid absorption phase was observed like other administrating routes (subcutaneous or intramuscular) and *t*_max_ of 0.24 ± 0.04 h in this work is in agreement with 0.3 h in mice [23] and 0.25 h in rabbits in previous report [24]. The elimination half-life was 0.44 ± 0.09 h, similar to previous study of 0.37 h, revealing a fast eliminating from blood circulation system [23]. A similar PK profiles of healthy quarter, infected quarter and suspected quarter were reported in previous literature, by which a negligible influence of the udder environment was claimed [12].

Secondly, in this work, the inoculum amounts of *S*. *aureus* strains were large enough to imitate the acute and severe intramammary infection, which was approximate of 7.5~8 logCFU/gland right before the drug administration. The initial massive bacterial load increased the burden of antibacterial activity of cefquinome, as a result dosages of 100 μg/gland or lower can only inhibit the growth of bacterium (slightly change in CFU) rather than killing of any (reduction of bacteria counts) at 24 h. In the 400 and 200 μg/gland single dose groups, 2.88 and 2.4 logCFU/gland differences of bacterial counts were observed at 24 h, respectively. Nevertheless, the antibacterial effects were similar (P > 0.05, two-tailed t-test). In addition, a single dose of 200 or 400 μg/gland cannot inhibit the bacterial regrowth after 72 h following administration. Thus, we can tell that it is difficult to achieve a bactericidal activity of 99.9% reduction of total bacteria *in vivo*, as the condition *in vivo* was much more complex than in culture medium. *In vivo*, drug concentration was ever changing and distribution of cefquinome was mainly in the extracellular fluid. But survival in both extracellular and intracellular were observed for *S*. *aureus* strains [25]. On the other hand, Staphylococcal infections do not always readily respond to antibiotic treatment and the pathogen can survive in the host in an attenuated form called small-colony variants, against which many antibiotics are not effective even they can penetrate mammalian cells [26].

To explore as widely as possible the potential clinical range, concentration range and therapeutic range in clinical situation, 21 regimens comprised of 7 doses and 3 dose intervals are investigated. From previous researches, the killing characteristic of cefquinome is time-dependent [8,23], and there is no doubt that %T>MIC is an essential parameter to describe the antibacterial activity with *R*^*2*^ of 0.8466.

However, an interesting outcome drew our attention. The influence of AUC_0-24_/MIC on treatment effectiveness is distinguished and considerable as well as the %T>MIC. For example, in time course killing trials, the *in vivo* antibacterial pattern of cefquinome has changed and been different from that *in vitro* (Figs 1 and 3). As the bolus dose increasing, the bacterial counts in gland tissue has dropped after 24 h observation, which suggests a dose related killing activity *in vivo*. Although the *R*^*2*^ of 0.908 for AUC/MIC is greater than %T>MIC numerically, the statistical significance evaluation is not available. Similarly, a dose-dependent manner of bacterial counts reduction in gland was observed following intramuscular and intravenous injections of cephapirin in the treatment of mouse mastitis [15]. Otherwise, the parameter of AUC/MIC is utilized to represent the pattern of antibacterial activity of time-dependent killing and prolonged persistent effect [27], and it combines both time and drug concentration factors with bacterial killing efficacy. In addition, the diffusion of cefquinome between blood and mammary gland, known as the blood-milk barrier, is limited. Drug distribution between blood and gland may not be identical when being compared to that between blood and thigh or among blood and lung tissues. Due to these reasons, the %T > MIC and AUC_0-24_/MIC are considered both to be important for the antibacterial efficacy.

It is not uncommon that an antibiotic drug’s PK/PD parameter could be more than one. For example, levofloxacin therapy in pulmonary, soft tissue, and urinary infections, two PK/PD parameters of C_max_/MIC and AUC/MIC were found to be essential predictors for its therapeutic effect [28]. In the present work, the significance of AUC/MIC index has elevated and both %T > MIC and AUC_0-24_/MIC are important following intramammary administration. Although non-target animal studies are not able to directly define the optimal clinical dose regimen when considering the species difference, they are still capable of defining the magnitude of the PK/PD index required for different treatment outcomes since various animal species including human should share a similar magnitude of the PK/PD index [27,29].

## Conclusions

To our knowledge, this is the first study applying a PK/PD model in IMI treatment. The activity of cefquinome against *S*. *aureus* was investigated through a mouse mastitis model. In conclusion, according to the integration of PK/PD, the parameters of %T>MIC and AUC_0-24_/MIC are important and may be mainly responsible for the prediction of antibacterial efficacy and the treatment outcomes of cefquinome after intramammary administration. Moreover, clinical dosing regimens may satisfy the %T>MIC and AUC_0-24_/MIC equals to or exceeds the value of 35.98% and 137.43 h so as to achieve a good antibacterial effect against *S*. *aureus* strains with an MIC of 0.5 μg/ml. Understanding the complexity of across species extrapolation of PK/PD data, the present study should be regarded as work providing rational understanding and some essential data for PK/PD evaluation aiming at optimizing bovine mastitis treatment strategy via intramammary drug administration.

## Supporting Information

S1 Fig *In vivo* growth curve in mouse model of *S*. *aureus* mastitis. (TIF)

S2 Fig The Bacterial colony count (log_10_ CFU/mL) in mammary gland following treatments of 21 dosing regimens. The dose ranged from 12.5 to 800 μg/gland and dosing intervals were 8, 12, and 24 h.
(TIF)

S1 Table MICs of cefquinome against 38 clinical isolates. (DOC)

S2 Table PK/PD parameters of 21 regimens following intramammary administration. (DOCX)

## References

[1] Gruet P, Maincent P, Berthelot X, Kaltsatos V (2001) Bovine mastitis and intramammary drug delivery: review and perspectives. Adv Drug Deliv Rev 50: 245–259. 11500230 10.1016/s0169-409x(01)00160-0

[2] Kirkan S, Goksoy EO, Kaya O (2005) Identification and antimicrobial susceptibility of Staphylococcus aureus and coagulase negative staphylococci from bovine mastitis in the Aydin region of Turkey. Turkish Journal of Veterinary & Animal Sciences 29: 791–796.

[3] Baranski W, Ras M, Janowski T, Zdunczyk S, Dewulf J, Kruif A, et al (2008) Udder pathogens isolated from milk of cows before drying off and their antibiotic sensitivity. Medycyna Weterynaryjna 64: 301–305.

[4] Intorre L, Vanni M, Meucci V, Tognetti R, Cerri D, Turchi B, et al (2013) Antimicrobial resistance of Staphylococcus aureus isolated from bovine milk in Italy from 2005 to 2011. Large Animal Review 19: 287–291.

[5] Al-Taher AY (2010) Pharmacokinetics of Cefquinome in Camels. J Anim Vet Adv 9: 848–852.

[6] Dinakaran V, Dumka VK, Ranjan B, Balaje R, Sidhu PK (2013) Pharmacokinetics following intravenous administration and pharmacodynamics of cefquinome in buffalo calves. Tropical Animal Health and Production 45: 1509–1512. 10.1007/s11250-013-0390-7 23456794

[7] Dumka VK, Dinakaran V, Ranjan B, Rampal S (2013) Comparative pharmacokinetics of cefquinome following intravenous and intramuscular administration in goats. Small Ruminant Research 113: 273–277.

[8] Shan Q, Yang F, Wang J, Ding H, He L, Zeng Z. (2014) Pharmacokinetic/pharmacodynamic relationship of cefquinome against Pasteurella multocida in a tissue-cage model in yellow cattle. J Vet Pharmacol Ther 37: 178–185. 10.1111/jvp.12076 23980645

[9] Uney K, Altan F, Elmas M (2011) Development and validation of a high-performance liquid chromatography method for determination of cefquinome concentrations in sheep plasma and its application to pharmacokinetic studies. Antimicrob Agents Chemother 55: 854–859. 10.1128/AAC.01126-10 21098247 PMC3028820

[10] Ehinger AM, Schmidt H, Kietzmann M (2005) Tissue distribution of cefquinome after intramammary and 'systemic' administration in the isolated perfused bovine udder. J Vet Pharmacol Ther 26: 93–93. 10.1016/j.tvjl.2005.02.029 16772139

[11] Cagnardi P, Gallo M, Zonca A, Locatelli C, Moroni P, Carli S, et al (2009) Pharmacokinetics of cefquinome in lactating cows after intramammary administration in healthy and infected animals. J Vet Pharmacol Ther 32: 145–146.

[12] Zonca A, Gallo M, Locatelli C, Carli S, Moroni P, Villa R, et al (2011) Cefquinome sulfate behavior after intramammary administration in healthy and infected cows. J Dairy Sci 94: 3455–3461. 10.3168/jds.2010-4109 21700031

[13] Hobarth G, Winter P, Baumgartner W (2004) Efficacy of cefquinome for-the treatment of bovine mastitis—a field study. Tierarztliche Umschau 59: 718-+.

[14] Bradley AJ, Breen JE, Payne B, Green MJ (2011) A comparison of broad-spectrum and narrow-spectrum dry cow therapy used alone and in combination with a teat sealant. J Dairy Sci 94: 692–704. 10.3168/jds.2010-3192 21257038

[15] Brouillette E, Grondin G, Lefebvre C, Talbot BG, Malouin F (2004) Mouse mastitis model of infection for antimicrobial compound efficacy studies against intracellular and extracellular forms of Staphylococcus aureus. Veterinary Microbiology 101: 253–262. 15261998 10.1016/j.vetmic.2004.04.008

[16] Brouillette E, Malouin F (2005) The pathogenesis and control of Staphylococcus aureus-induced mastitis: study models in the mouse. Microbes Infect 7: 560–568. 15777742 10.1016/j.micinf.2004.11.008

[17] Newbould FH (1974) Antibiotic treatment of experimental Staphylococcus aureus infections of the bovine mammary gland. Can J Comp Med 38: 411–416. 4279760 PMC1319843

[18] Institute of Laboratory Animal Research, Commission on Life Sciences, National Research Council (1996) Guide for the care and use of laboratory animals: National Academy Press, Washington, DC.

[19] CLSI (2013) *Performance Standards for Antimicrobial Disk and Dilution Susceptibility Tests for Bacterial Isolated from Animals; Approved Standard-Fourth Edition and Supplement*, VET01A4E and VET01S2E PA: Clinical and Laboratory Standards Institute.

[20] Schwarz S, Silley P, Simjee S, Woodford N, van Duijkeren E, Johnson AP, et al (2010) Editorial: assessing the antimicrobial susceptibility of bacteria obtained from animals. Journal of antimicrobial chemotherapy: dkq037. 10.1093/jac/dkq037 20181573

[21] Zhou YF, Zhao DH, Yu Y, Yang X, Shi W, Peng YB, et al (2015) Pharmacokinetics, bioavailability and PK/PD relationship of cefquinome for Escherichia coli in Beagle dogs. J Vet Pharmacol Ther 38: 543–548. 10.1111/jvp.12225 25776615

[22] Zhao DH, Zhou YF, Yu Y, Shi W, Yang X, Xiao X, et al (2014) Integration of pharmacokinetic and pharmacodynamic indices of valnemulin in broiler chickens after a single intravenous and intramuscular administration. Vet J 201: 109–115. 10.1016/j.tvjl.2014.05.010 24906499

[23] Wang J, Shan Q, Ding H, Liang C, Zeng Z (2014) Pharmacodynamics of cefquinome in a neutropenic mouse thigh model of Staphylococcus aureus infection. Antimicrob Agents Chemother 58: 3008–3012. 10.1128/AAC.01666-13 24614373 PMC4068449

[24] Hwang YH, Song IB, Lee HK, Kim TW, Kim MS, Lim JH, et al (2011) Pharmacokinetics and bioavailability of cefquinome in rabbits following intravenous and intramuscular administration. J Vet Pharmacol Ther 34: 618–620. 10.1111/j.1365-2885.2011.01289.x 21615754

[25] Limbert M, Isert D, Klesel N, Markus A, Seeger K, Seibert G, et al (1991) Antibacterial activities in vitro and in vivo and pharmacokinetics of cefquinome (HR 111V), a new broad-spectrum cephalosporin. Antimicrob Agents Chemother 35: 14–19. 2014969 10.1128/aac.35.1.14 PMC244934

[26] Malouin F, Brouillette E, Martinez A, Boyll BJ, Toth JL, Gage JL, et al (2005) Identification of antimicrobial compounds active against intracellular Staphylococcus aureus. FEMS Immunol Med Microbiol 45: 245–252. 15963705 10.1016/j.femsim.2005.04.003

[27] Andes D, Craig WA (2002) Animal model pharmacokinetics and pharmacodynamics: a critical review. Int J Antimicrob Ag 19: 261–268. 10.1016/s0924-8579(02)00022-5 11978497

[28] Andes D, Craig WA (2002) Pharmacodynamics of the new fluoroquinolone gatifloxacin in murine thigh and lung infection models. Antimicrob Agents Chemother 46: 1665–1670. 12019073 10.1128/AAC.46.6.1665-1670.2002 PMC127205

[29] Craig WA (1998) Pharmacokinetic/pharmacodynamic parameters: rationale for antibacterial dosing of mice and men. Clin Infect Dis 26: 1–10; quiz 11–12. 9455502 10.1086/516284

