# Supplementary figures and images for: In Vivo Pharmacokinetics/Pharmacodynamics of Cefquinome in an Experimental Mouse Model of Staphylococcus Aureus Mastitis following Intramammary Infusion
Source: PLoS One. 2016 May 24;11(5):e0156273. doi: 10.1371/journal.pone.0156273 (PMC4878769; doi:10.1371/journal.pone.0156273)

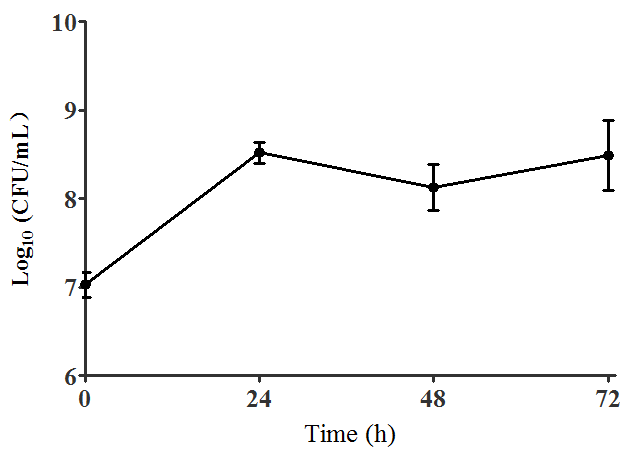

Supplement: S1 Fig — (TIF) [file pone.0156273.s001.tif]

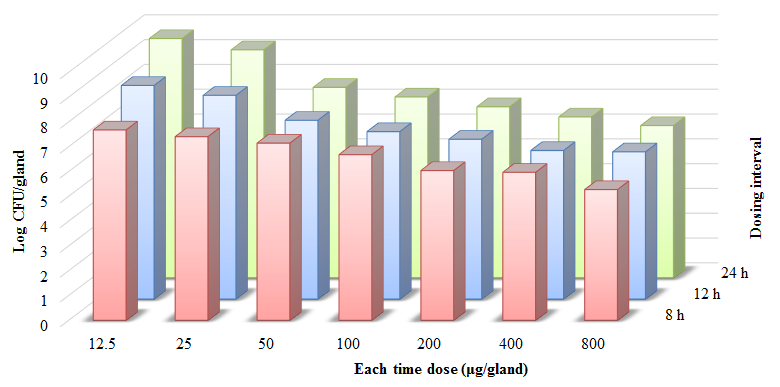

Supplement: S2 Fig — The dose ranged from 12.5 to 800 μg/gland and dosing intervals were 8, 12, and 24 h. (TIF) [file pone.0156273.s002.tif]
