# Supplementary material for: In Vivo Pharmacokinetics/Pharmacodynamics of Cefquinome in an Experimental Mouse Model of Staphylococcus Aureus Mastitis following Intramammary Infusion
Source: PLoS One. 2016 May 24;11(5):e0156273. doi: 10.1371/journal.pone.0156273 (PMC4878769; doi:10.1371/journal.pone.0156273)
Supplement: S1 Table — (DOC) [file pone.0156273.s003.doc]

***In vivo* Pharmacokinetics/Pharmacodynamics of Cefquinome in an experimental Mouse Model of *Staphylococcus Aureus* Mastitis Following Intramammary Infusion**

Yang Yu^1,2^, Yu-Feng Zhou^1,2^, Mei-Ren Chen^1,2^, Xiao Li^1,2^, Gui-Lin Qiao^3^, Jian Sun^1,2^, Xiao-Ping Liao^1,2^, Ya-Hong Liu^1,2,*^

**S1 Table.** MICs of cefquinome against 38 clinical isolates

| **Isolates** | **MIC** |  |
| --- | --- | --- |
| NF140151JP | 0.25 |  |
| NF140162JP | 0.25 |  |
| NF140172JP | 0.25 |  |
| NH140633JP | 0.25 |  |
| NH140683JP | 0.25 |  |
| NH140703JP | 0.25 |  |
| NH140713JP | 0.25 |  |
| NH140723JP | 0.25 |  |
| NH140911JP | 0.25 |  |
| NH140793JP | 0.25 |  |
| NH140743JP | 0.25 |  |
| NW140394JP | 0.25 |  |
| NF140264JP | 0.5 |  |
| NH140074JP | 0.5 |  |
| NF140251JP | 0.5 |  |
| NF140252JP | 0.5 |  |
| NF140263JP | 0.5 |  |
| NF140181JP | 0.5 | MIC_50_ |
| NF140204JP | 0.5 |  |
| NF140192JP | 0.5 |  |
| NH140572JP | 0.5 |  |
| NH140702JP | 0.5 |  |
| NH140751JP | 0.5 |  |
| NH140752JP | 0.5 |  |
| NH140842JP | 0.5 |  |
| NH140831JP | 0.5 |  |
| NH140802JP | 0.5 |  |
| NH140821JP | 0.5 |  |
| NH140792JP | 0.5 |  |
| NH140771JP | 0.5 |  |
| NH140742JP | 0.5 |  |
| NH140574JP | 0.5 |  |
| NW140312JP | 0.5 | MIC_90_ |
| NW140404JP | 0.5 |  |
| NH140851JP | 0.5 |  |
| NH140862JP | 0.5 |  |
| NH140871JP | 0.5 |  |
| NH140634JP | 0.5 |  |

**S2 Table.** PK/PD parameters of 21 regimens following intramammary administration.

| **Dosage** | **Dose Intervals (h)** | **AUC/MIC (h)** | **%T>MIC** | **C_max_/MIC** |
| --- | --- | --- | --- | --- |
| 12.5 | 8 | 3.29 | 6.10 | 1.24 |
|  | 12 | 2.19 | 4.07 | 1.24 |
|  | 24 | 1.10 | 2.03 | 1.24 |
|  |  |  |  |  |
| 25 | 8 | 6.09 | 10.96 | 2.47 |
|  | 12 | 4.06 | 7.31 | 2.47 |
|  | 24 | 2.03 | 3.65 | 2.47 |
|  |  |  |  |  |
| 50 | 8 | 12.19 | 15.83 | 4.95 |
|  | 12 | 8.12 | 10.55 | 4.95 |
|  | 24 | 4.06 | 5.28 | 4.95 |
|  |  |  |  |  |
| 100 | 8 | 24.37 | 20.70 | 9.89 |
|  | 12 | 16.25 | 13.80 | 9.89 |
|  | 24 | 8.12 | 6.90 | 9.89 |
|  |  |  |  |  |
| 200 | 8 | 48.74 | 25.56 | 19.78 |
|  | 12 | 32.50 | 17.04 | 19.78 |
|  | 24 | 16.25 | 8.52 | 19.78 |
|  |  |  |  |  |
| 400 | 8 | 97.49 | 30.43 | 39.57 |
|  | 12 | 64.99 | 20.28 | 39.57 |
|  | 24 | 32.50 | 10.14 | 39.57 |
|  |  |  |  |  |
| 800 | 8 | 194.97 | 35.29 | 79.13 |
|  | 12 | 129.98 | 23.53 | 79.13 |
|  | 24 | 64.99 | 11.76 | 79.13 |
